# Population pharmacokinetics and pharmacodynamics of hydroxyurea in sickle cell anemia patients, a basis for optimizing the dosing regimen

**DOI:** 10.1186/1750-1172-6-30

**Published:** 2011-05-28

**Authors:** Ines Paule, Hind Sassi, Anoosha Habibi, Kim PD Pham, Dora Bachir, Frédéric Galactéros, Pascal Girard, Anne Hulin, Michel Tod

**Affiliations:** 1 Université de Lyon, Lyon, France; 2 EMR3738 CTO, Faculté de Médecine Lyon-Sud, Université Lyon 1, Oullins, France; 3 Laboratoire de Pharmacologie, AP-HP, GH H. Mondor, Université Paris Est-Créteil, Créteil, France; 4 Centre de référence pour les syndromes drépanocytaires majeurs, AP-HP, GH H. Mondor, Université Paris Est-Créteil, Créteil, France; 5 Hôpital Croix-Rousse, Hospices Civils de Lyon, Lyon, France

## Abstract

**Background:**

Hydroxyurea (HU) is the first approved pharmacological treatment of sickle cell anemia (SCA). The objectives of this study were to develop population pharmacokinetic(PK)-pharmacodynamic(PD) models for HU in order to characterize the exposure-efficacy relationships and their variability, compare two dosing regimens by simulations and develop some recommendations for monitoring the treatment.

**Methods:**

The models were built using population modelling software NONMEM VII based on data from two clinical studies of SCA adult patients receiving 500-2000 mg of HU once daily. Fetal hemoglobin percentage (HbF%) and mean corpuscular volume (MCV) were used as biomarkers for response. A sequential modelling approach was applied. Models were evaluated using simulation-based techniques. Comparisons of two dosing regimens were performed by simulating 10000 patients in each arm during 12 months.

**Results:**

The PK profiles were described by a bicompartmental model. The median (and interindividual coefficient of variation (CV)) of clearance was 11.6 L/h (30%), the central volume was 45.3 L (35%). PK steady-state was reached in about 35 days. For a given dosing regimen, HU exposure varied approximately fivefold among patients. The dynamics of HbF% and MCV were described by turnover models with inhibition of elimination of response. In the studied range of drug exposures, the effect of HU on HbF% was at its maximum (median I_max _was 0.57, CV was 27%); the effect on MCV was close to its maximum, with median value of 0.14 and CV of 49%. Simulations showed that 95% of the steady-state levels of HbF% and MCV need 26 months and 3 months to be reached, respectively. The CV of the steady-state value of HbF% was about 7 times larger than that of MCV. Simulations with two different dosing regimens showed that continuous dosing led to a stronger HbF% increase in some patients.

**Conclusions:**

The high variability of response to HU was related in part to pharmacokinetics and to pharmacodynamics. The steady-state value of MCV at month 3 is not predictive of the HbF% value at month 26. Hence, HbF% level may be a better biomarker for monitoring HU treatment. Continuous dosing might be more advantageous in terms of HbF% for patients who have a strong response to HU.

**Trial Registration:**

The clinical studies whose data are analysed and reported in this work were not required to be registered in France at their time. Both studies were approved by local ethics committees (of Mondor Hospital and of Kremlin-Bicetre Hospital) and written informed consent was obtained from each patient.

## Disease name

Sickle cell anemia.

## Definition

Sickle cell anemia is an autosomal recessive genetic blood disorder, caused by a mutation in the hemoglobin gene and characterized by rigid sickle-shaped red blood cells. Sickling decreases the cells' elasticity and leads to vaso-occlusion which may result in various complications, such as acute painful crises, ischemia and damage of various organs, acute chest syndrome or stroke.

## Background

The antineoplastic agent hydroxyurea (hydroxycarbamide) (HU) is the first approved pharmacological treatment of sickle cell anemia (SCA). It inhibits the production of the hemoglobin S that causes SCA and favors the reactivation of fetal hemoglobin (HbF) expression [1]. In fact, a variety of mechanisms are believed to be involved in HU beneficial effects in SCA, including increased HbF synthesis by erythroid regeneration, NO-related increases in soluble guanylate cyclase activity and cyclic guanidine monophosphate (cGMP) that stimulate *HBG *expression [2]. Other mechanisms may be myelosuppression with a reduction of circulating neutrophils, increased erythrocyte water content, modified erythrocyte endothelial cell interactions and altered vascular tone by increasing NO bioavailability [3]. Recently, Bartolucci *et al. *reported that HU could reduce abnormal sickle cell adhesion to the vascular wall by regulating the activation state of adhesion molecules [4]. HU treatment reduces the rate and severity of painful attacks [5] and was shown to possibly increase survival time [6].

The usual dosing of this oral treatment is daily doses of 15-35 mg/kg (or less if there is renal insufficiency) [7]. The most adequate individual dose is determined by starting with 15 mg/kg and monitoring full blood cell counts every two weeks. If after twelve weeks no cytopenia has developed, the dose is increased by 5 mg/kg. Once their maximal tolerable dose is determined, the patient can continue the treatment life-long, if no serious toxicities manifest or other issues arise.

Despite the widespread use of HU, only a few studies have been reported in the literature, especially concerning its use in the indication of SCA. Little is known about the relationship between drug exposure and efficacy, evaluated by fetal hemoglobin (HbF) and mean corpuscular volume (MCV) measurements. Although a number of genetic polymorphisms have been found to be associated with response to HU [8-10], the variability of this response remains poorly characterized. The optimal dosing schedule, the best strategy for monitoring and adjusting the treatment, and the impact that may have the prior determination of candidate genotypes on HU dosing remain open to discussion. Part of these questions may be addressed through simulations from pharmacokinetic (PK) and pharmacodynamic (PD) models of HU. Therefore, this study aimed to develop population PK-PD models for HU in order to characterize the exposure-efficacy relationships and their variability. These models were then used (1) to compare two dosing regimens (one continuous daily and the other with interruptions of 2 days after every 5 days) by simulation, and (2) to develop some recommendations for monitoring the treatment.

## Methods

Two datasets from two studies with different designs were used in this analysis: one from a PK-PD study with up to 9 samples taken per patient over a period of up to 30 months, and the other from a PK study with 10 samples per patient taken over 24 hours after drug administration.

### PKPD study design (sparse sampling)

#### Study design and patient population

This 30-month, open-label, noncomparative, prospective, observational study was conducted in 81 adult patients with sickle cell anemia at the *Centre de référence pour les syndromes drépanocytaires majeurs*, AP-HP, GH H. Mondor, *Université Paris Est-Créteil*, France from 2007 to 2010. It focused on the benefits and risks of HU, in particular the side effects in the short and medium term, as well as the need for regular hematological monitoring. The protocol was approved by the local ethics committee of Mondor hospital, and written informed consent was obtained from each patient. The recruited SCA patients were of 18 years or older and with Hb genotype *HbSS *(homozygous sickle Hb). In all patients, SCA diagnosis was documented by standard methods [7]. Patients who received erythropoietin or a blood transfusion at a time that can interfere with the results were excluded.

#### Treatment

HU treatment was started at a dose of 20 mg/kg orally unless there was renal insufficiency (in this case it started at 10 and 15 mg/kg). Since hematotoxicity is the dose-limiting toxicity, the hematologic control was performed every 15 days during the first month and then every month; if needed, doses were adjusted to maintain neutrophil counts higher than 3 × 10^9^/L. In most cases, the final dose of HU did not exceed 30 mg/kg.

#### Pharmacokinetics protocol

Blood was drawn from most patients on day 0 (D0), D15, after 1 month (M1), M2, M4 and M6, as well as at various later timepoints more sparsely (up to M30). It was collected in heparinized tubes and centrifuged at 2000 g for 10 minutes at room temperature to obtain plasma. The plasma was then stored at -20°C until the samples were assayed. Plasma samples were assayed using high performance liquid chromatography (HPLC) coupled with UV-detection at 449 nm [11]. The analytical method was linear between 5 to 1000 μM, precise (coefficients of variation ranging from 1.7 to 9.9%), accurate (97.7 to 103.9%). The limit of quantification (LOQ) was 7 μM (0.532 mg/L).

The following biologic variables were measured during this study: creatinine, urea, lactate dehydrogenase (LDH), hemoglobin (Hb), fetal hemoglobin (HbF), mean corpuscular volume (MCV), mean corpuscular hemoglobin (MCH), ferritin, bilirubin, aspartate transaminase (AST), alanine transaminase (ALT), neutrophils (PMN) and platelets. Body weight, age and sex data were also documented.

### PK study design (rich sampling)

These rich PK data come from a bioequivalence study of standard hydroxyurea capsules and a new formulation of 1000 mg coated breakable tablets in adults with homozygous SCA or S/β-thalassemia. The complete study protocol is given in a publication of the noncompartmental analysis of the PK data [12]. The assay validation parameters were the same as for the sparse PK data study [11].

These data contain 10 blood samples per patient, from 16 patients who took hydroxyurea doses ranging from 1000 mg to 2000 mg. The samples were taken at baseline and 45, 90, 120, 150, 180, 240, 360, 480 minutes after HU administration at the study center, as well as trough samples after 24 hours.

### Population PK-PD models

Population analyses were performed using NONMEM software (version VII) [13]. PK model parameters were estimated using the second order conditional estimation (Laplacian) method with interaction between interindividual and residual variabilities. This estimation method is more accurate than the standard first order conditional estimation (FOCE) method. In this PK analysis it was needed to correctly handle the concentrations below the limit of quantification (BLQ) (see below). PD models parameters were estimated using the FOCE method with interaction between interindividual and residual variabilities. Confidence intervals (CI) of parameter estimates were obtained by nonparametric bootstrap (n = 1000) with stratification by study and dose.

#### Population pharmacokinetic model

First, the structural PK model was built using both sparse and rich datasets. One, two and three-compartment models with first-order absorption and elimination were tested, as well as with saturable (Michaelis-Menten) elimination. The most appropriate model was chosen on the basis of the objective function value (OFV) and simulation-based diagnostics such as normalised predictive discrepancy errors (NPDE) [14]. The NPDE analysis was performed with BLQ points excluded.

In the two-compartment model, the parameters were the elimination clearance (CL/F), the volume of the central compartment (V_c_/F), the rate constants for transfer from central to peripheral (k_cp_) or peripheral to central compartment (k_pc_), the rate constant for absorption (k_a_).

The BLQ measurements of the sparse data were included in the data and modelled as censored observations using "method 3" described in [15].

The interindividual variability (IIV) for the PK parameters was described using exponential models: , where η is a random variable with normal distribution, zero mean and variance to be estimated, θ is a typical value. The IIV was added in a stepwise manner, firstly to clearance and central volume of distribution. The interindividual random effects were kept in the model if their inclusion significantly reduced the OFV and if their relative SE was <50%. A full interindividual variance-covariance matrix was estimated to assess if there was any significant covariance in the IIV structure.

To find the most appropriate residual error model, additive, proportional and mixed error models were tested. As data were collected from two quite differently designed studies, separate residual error models for each study were tested.

The variables that were investigated for their ability to explain IIV in the PK of HU were body weight, creatinine, age and sex. Scaling by body weight (for clearance and central volume of distribution) as  and , as well as  (with sex = 0 for men and 1 for women, *coeff*_*sex *_estimated), similar to the Cockroft-Gault formula for creatinine clearance [16], were the tested forms of relationships. The conditions for the variable to be retained as a covariate were to be biologically plausible, and to decrease the OFV by at least 5 units, corresponding to a p-value less than 2.5%.

Missing values of body weight were predicted by the model: if the patient's weight was known at other timepoint(s), it was predicted by using an interpolated or adjacent value and an additive individual random effect whose variance was fixed to the observed intraindividual variance of the body weight of the dataset (1.5); if no data of patient's weight were available, it was predicted by using the average weight of the dataset (62 kg for women, 65 kg for men) and an additive individual random effect whose variance was fixed to the observed interindividual variances of the dataset (92 for women, 40 for men).

The time to reach 95% of HU concentration steady-state was determined by simulation of a typical patient with 1000 mg dose every day for 6 months.

#### Population pharmacodynamic models

The PD responses to be described by the model were HbF percentage and MCV. The observed level of both these responses depends on the ratio of a production rate to an elimination rate. Therefore, turnover models [17] were chosen to fit the data. Models assuming either stimulation of the production or inhibition of the elimination of response by HU were tested. The metrics of HU exposure used as the input into the PD model was mean drug concentration at steady state, calculated using the individual posthoc estimates of HU clearance. Therefore, a sequential modelling approach was used. The uncertainty in the estimate of clearance was taken into account by using the IPPSE method [18]. Linear, Emax, sigmoid and power functions were tested for the effect of the drug. The final model was chosen on the basis of the OFV and diagnostic plots, stability and precision of parameter estimates. IIV and residual error models were constructed in the same way as for the PK model.

In addition to demographic variables, baseline measurements of pharmacodynamic variables, as well as their previous values, rate of change per day from baseline to the previous value (), rate of change per day between the two previous values () were investigated as covariates. They were included as additive or proportional to the drug effect, production rate or elimination rate. Missing values were predicted by the model by using the data average and an individual random effect whose variance was fixed to the value calculated from the data. The conditions for covariate inclusion were the same as for the PK model.

#### Model evaluation

The quality of models was assessed by goodness-of-fit plots and simulation-based methods (using 1000 simulations): visual predictive check (VPC) and NPDE. The mean of prediction error distribution was compared to zero by a Wilcoxon signed rank test, while its variance was compared to unity by a Fisher test. For the VPC, prediction corrections were used so that data of all dose levels could be used in one plot [19]. BLQ points in the observed and simulated PK data sets were assigned values equal to LOQ/2. The VPC plots showed 80% prediction intervals (PI) and medians of the observed and of the predicted data, as well as 95% confidence areas around the percentiles. For the PK, a VPC plot in log scale was also given.

### Simulation of alternative dosing regimens

The two simulated dosing regimens were: 1000 mg daily doses 7 days a week (7/7) and 1000 mg daily doses 5 consecutive days a week (5/7) for the duration of 12 months; 10000 patients were simulated in each arm. For each covariate, the average observed value was used. The results were compared graphically by representing the median profile and the 90% prediction interval of the HbF% and MCV. In order to determine the steady-state values of the HbF% and of the MCV and time to reach 95% of them, such simulations with 7/7 dosing were extended for 48 months.

## Results

### Pharmacokinetic data analysis

A summary of PK related patient characteristics is given in Table 1. In the sparse data, 78% of last doses before the concentration measurements were 1000 mg, 15% were 1500 mg, and the rest were 500 mg, 1250 mg or 2000 mg. In the rich dataset, 44% were 1000 mg, 31% were 1500 mg, and the rest were either 1250 mg or 2000 mg. The median number of measurements per patient in the sparse dataset was 4 (range: 1 - 9); in the rich dataset, 10 measurements were available for each patient.

**Table 1:** Summary of patient characteristics

	PKPD dataset	Rich PK dataset
**Characteristics**	**Median (range)**	**Median (range)**

Age	30 (18 - 54)	32 (24 - 52)
Men/women	24/57	5/11
Weight (kg)	60 (45 - 163)	63 (42 - 71)
Creatinine (μM)	65 (27 - 558)	72 (47 - 129)
Urea (μM)	3.1 (0.8 - 10.5)	3.4 (2.4 - 7.7)

The PK profiles were best described by a two-compartment model (with first-order absorption and elimination). The NPDE diagnostics indicated that one-compartment model could not adequately describe these data. The OFV of the three-compartment model was not lower than that of the two-compartment model. No significant nonlinearity in absorption or elimination could be detected in these data.

Significant interindividual variability was found for V_c_, CL, k_a _and k_cp_. Correlations were significant among the individual values of V_c_, CL and k_cp_. The mixed residual error model was best for both datasets. The OFVs of PK models with and without weight-scaling of V_c _and CL (as shown in methods section) were nearly significant, but this covariate was kept in the model for the sake of coherence with clinical practice and possible application of the model to children (where the effect of weight would be much more perceptible). The allometric model for clearance with power 0.75 was better than the model with power 1 (p = 0.0016).

The final model estimates are given in Table 2. As the bioavailability was not estimated here, the reported estimates for CL and V_c _represent apparent pharmacokinetic parameters CL/F and V_c_/F. Their values in Table 2 are given for a patient of 70 kg weight, which is the scaling base. To obtain values for patients of different weight, the population value should be multiplied by weight/70 for V_c _and by (weight/70)^0.75 ^for CL. The estimate of the rate constant of transfer from the peripheral to the central compartment (k_pc_) was very small and unstable, so its value was fixed to 0.004 (h^-1^) (its best estimate) and this resulted in lower estimate of IIV of k_cp _and better stability of the model. With this model, the time to reach 95% of the steady-state was typically about 35 days.

**Table 2:** Parameter estimates of the population PK model

Parameters	Typical values (95% CI)	Standard deviations of IIV (95% CI)	Interindividual CV
V_c_/F (L) (for a patient of 70 kg)	45.3 (38.9 - 50.5)	0.34 (0.23 - 0.46)	35%
Cl/F (L/h) (for a patient of 70 kg)	11.6 (10.4 - 12.9)	0.29 (0.22 - 0.40)	30%
k_a _(h^-1^)	3.29		
θ_ka _(h^-1^) ()	3.02 (2.25 - 4.19)	1.34 (1.16 - 1.65)	224%
k_cp _(h^-1^)	0.027 (0.021 - 0.037)	0.57 (0.43 - 0.95)	62%
k_pc _(h^-1^) (fixed)	0.004	-	-
SD of the additive component of the residual error (mg/L)			
- for densely sampled data	0.319 (0.197 - 0.492)		
- for sparsely sampled data	0.353 (0.257 - 0.522)		
SD of the proportional component of the residual error			
- for densely sampled data	0.12 (0.083 - 0.154)		
- for sparsely sampled data	0.435 (0.349 - 0.506)		
Correlation (η_Vc_, η_Cl_)	0.71		
Correlation (η_Vc_, η_kcp_)	-0.26		
Correlation (η_CL_, η_kcp_)	0.37		

CI: confidence intervals, obtained by bootstrap (n = 1000), IIV: interindividual variability, CV: the apparent coefficient of variation of interindividual variability.

**Figure 1:**
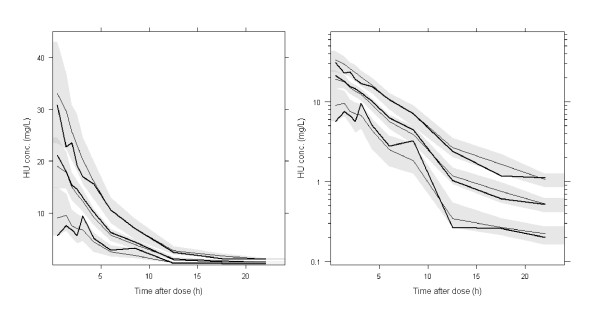
**PC-VPC for HU concentrations**. The lines show the 10^th^, 50^th ^and 90^th ^percentiles of observed data (thick lines) and of predictions (thin lines); the grey areas represent the 95% confidence areas around the percentiles. PC-VPC: prediction-corrected visual predictive check. Left panel: linear scale, right panel: log scale.

Simulation-based diagnostics VPC (Figure 1) and NPDE indicated that the model adequately described the measured data. The mean of normalized prediction errors was significantly different from zero (mean = 0.12, p = 0.016), but translated into HU concentrations, the mean difference between observed and predicted concentrations was only 0.08 mg/L. This difference was not considered as relevant from a clinical point of view. The variance of normalized prediction errors was not significantly different from unity (0.893, p = 0.12).

### Pharmacodynamic data analysis

**Figure 2:**
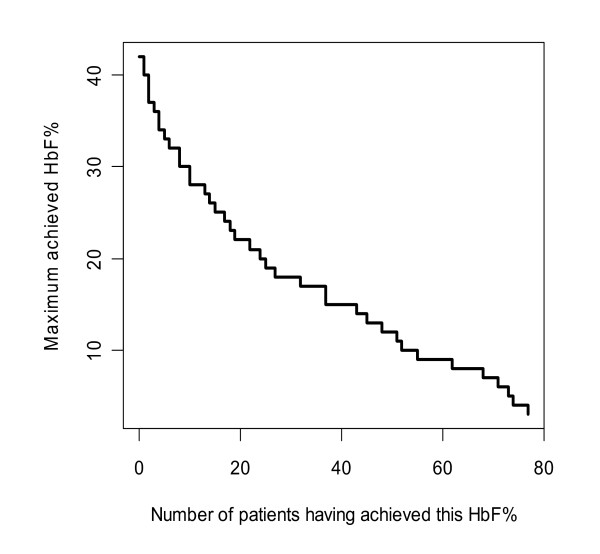
**Maximum achieved HbF% and corresponding cumulative number of patients**.

The median number of HbF% measurements per patient was 5 (range: 1 - 10); the total number of patients and measurements was 77 and 391 respectively. The median number of MCV measurements per patient was 6 (range: 3 - 10); the total number of patients and measurements was 80 and 439 respectively. 43% of patients with HbF% measurements and 49% of patients with MCV measurements were followed for 6 months (median (range) for HbF%: 6 months (9 days - 30 months); for MCV: 6 months (1 - 30 months)). Summary statistics of the pharmacodynamic variables at the beginning and after 6 months in the study are given in Table 3. The treatment with HU induced an increase in HbF, its percentage (Figure 2 shows its maximums) and in MCV, as well as decreases in bilirubin and LDH, which were indicative of decrease in the rate of hemolysis. Decreases in neutrophils (PMN) and platelets were mild, not reaching below normal levels.

**Table 3:** Summary statistics of PD variables

PD variables	At baseline Median (range)	Number of patients	After 6 months of treatment Median (range)	Number of patients
HbF%	6.3 (0.6 - 30.7)	65	15.7 (3.9 - 41.6)	46
HbF (g/dL)	0.48 (0.04 - 2.7)	63	1.59 (0.34 - 4.04)	46
Hemoglobin (g/dL)	8.8 (6.3 - 11.9)	73	9.6 (6.9 - 14.4)	55
MCV (fL)	90 (68 - 113)	74	111 (81 - 131)	55
MCH (pg)	30 (21 - 36)	73	37 (25 - 44)	55
PMN (10^9^/L)	56 (25 - 80)	71	49 (26 - 86)	53
Platelets (10^9^/L)	428 (122 - 995)	74	316 (109 - 528)	55
Bilirubin (μM)	43 (9 - 96)	75	30 (6 - 113)	53
LDH (UI/L)	355 (155 - 800)	73	317 (168 - 766)	52
Ferritin (μg/L)	346 (16 - 4500)	72	275 (14 - 2940)	52
AST (UI/L)	32 (17 - 79)	75	31 (12 - 81)	53
ALT (UI/L)	22 (7 - 84)	75	21 (7 - 83)	53
Creatinine (μM)	65 (38 - 142)	75	64 (35 - 137)	52
Urea (μM)	2.9 (1.2 - 13.3)	75	3.3 (1.5 - 9)	52

#### Population PD model of the percentage of fetal hemoglobin

The turnover model with inhibition of the elimination rate was found to describe best the HbF% data. However, in this dataset, no relationship between HU concentration and HbF% could be identified, because all patients were estimated to have the maximum drug effect. The rate of change per day between the two last MCV observations (ΔMCV) was found to be a significant covariate for the production rate, K_in _(p < 0.00001). The median (range) ΔMCV was 0.16 (-0.27 to 0.87). The final model was (cf. parameter estimates in Table 4):

**Table 4:** Parameter estimates of the population HbF% model

Parameters	Typical values (95% CI)	SD of IIV (95% CI)	Interindividual CV
K_in _(%/day)	0.071 (0.055 - 0.094)	0.585 (0.472 - 0.681)	63%
K_out _(day^-1^)	0.013 (0.010 - 0.019)	0.486 (0.334 - 0.602)	52%
L_Imax _(unitless)	0.276 (-0.081 - 0.644) (I_max _= 0.569 (0.48 - 0.656))	1.44 (1.07 - 1.97)	27%
*θ*_Δ*MCV *_(day^-1^)	1.37 (0.95 - 1.76)		
SD of proportional residual error	0.142 (0.119 - 0.162)		
Correlation (η_Kout_, η_Imax_)	0.892		

CI: confidence intervals, obtained by bootstrap (n = 1000), SD: standard deviation, IIV: interindividual variability, CV: the apparent coefficient of variation of interindividual variability.

where , ,  and L_Imax _is the logit-transformed Imax.

Significant interindividual variability was found for K_in_, K_out _and L_Imax_, with correlation between K_out _and L_Imax_. A proportional residual error model was selected.

**Figure 3:**
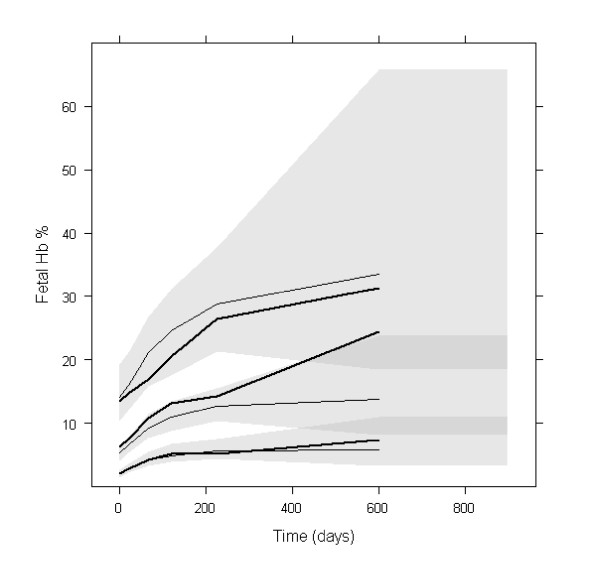
**PC-VPC for HbF%**. The lines show the10^th^, 50^th ^and 90^th ^percentiles of observed data (thick lines) and of predictions (thin lines); the grey areas represent the 95% confidence areas around the percentiles. PC-VPC: prediction-corrected visual predictive check.

Simulation-based diagnostics VPC (Figure 3) and NPDE indicated that the model adequately described the observed HbF% data. The mean of NPDE was 0.03 (p = 0.3), the variance was 0.937 (p = 0.38). Concerning the VPC, for the first 300 days, the difference between the medians of HbF% observations and of its predictions was approximately 2%, which was not clinically significant. Only 14 patients out of 81 continued the treatment longer than 300 days, therefore the percentiles at later times may be imprecise. The NPDE versus time plots did not indicate any prediction deficiencies (data not shown).

In the simulation, median HbF% at steady-state was about 18.6%, 95% of it was reached in about 26 months.

#### Population PD model of the mean corpuscular volume

The turnover model with inhibition of the elimination rate was found to describe best the MCV data. The inhibition was best described by a power function of average concentration. The rate of change per day between the two last HbF% observations (ΔHbF%) was found to be a significant covariate on the parameter β (p < 0.00001). The median (range) ΔHbF% was 0.047 (-0.278 to 0.653). The final model was (cf. parameter estimates in Table 5):

**Table 5:** Parameter estimates of the population MCV model

Parameters	Typical values (95% CI)	SD of IIV (95% CI)	Interindividual CV
K_in _(%/day)	3.71 (3.13 - 4.30)	0.191 (0.083 - 0.401)	19%
K_out _(day^-1^)	0.042 (0.035 - 0.048)	0.186 (0.044 - 0.415)	19%
β (L. mg^-1^)^1/γ^	0.099 (0.064 - 0.135)	0.457 (0.336 - 0.599)	48%
γ (unitless)	0.19 (0.02 - 0.46)		
*θ*_Δ*HbF% *_(day^-1^)	1.22 (-0.07 - 2.21)		
SD of proportional residual error	0.036 (0.030 - 0.040)		
Correlation (η_Kin_, η_Kout_)	0.87		
Correlation (η_Kin_, η_β_)	-0.98		
Correlation (η_Kout_, η_β_)	-0.95		

CI: confidence intervals, obtained by bootstrap (n = 1000), SD: standard deviation, IIV: interindividual variability, CV: the apparent coefficient of variation of interindividual variability.

where , , 

Significant interindividual variability was found for K_in_, K_out _and β, with correlations between all three parameters. A proportional residual error model was selected.

**Figure 4:**
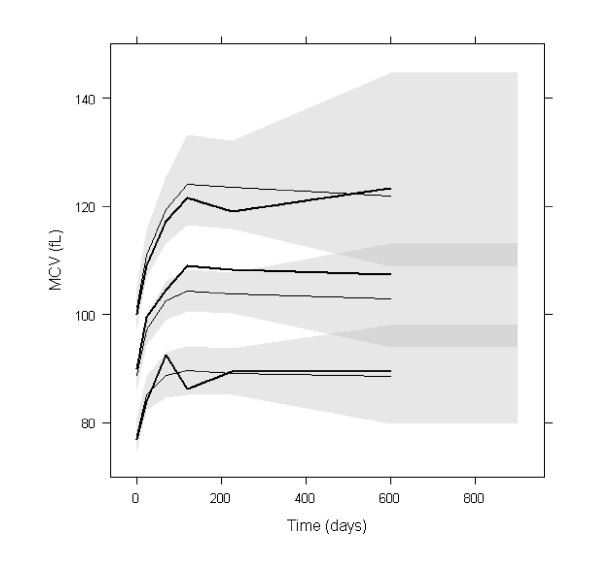
**PC-VPC for MCV**. The lines show the 10^th^, 50^th ^and 90^th ^percentiles of observed data (thick lines) and of predictions (thin lines); the grey areas represent the 95% confidence areas around the percentiles. PC-VPC: prediction-corrected visual predictive check.

Simulation-based diagnostics VPC (Figure 4) and NPDE indicated that the model adequately described the observed MCV data. The mean of NPDE was -0.02 (p = 0.87), the variance was 0.9 (p = 0.15). In the VPC, the difference between the medians of MCV observations and of its predictions was approximately 5 fL, which was not clinically significant. In the simulation, median MCV level at steady-state was about 104 pL, 95% of it was reached in about 90 days.

### Simulation of alternative dosing regimens

**Figure 5:**
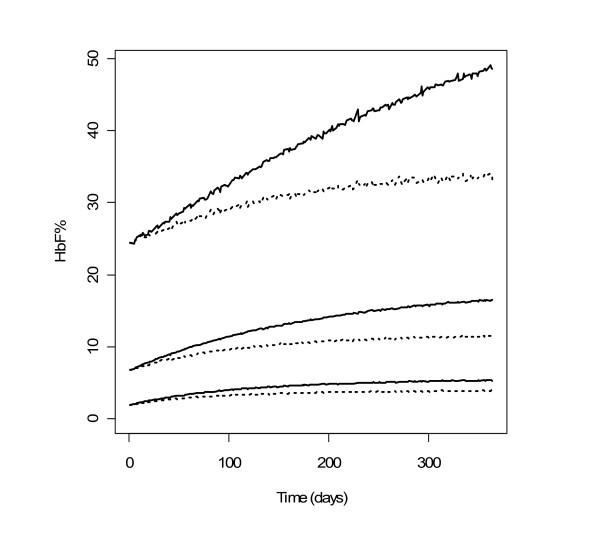
**Profiles of simulated HbF% with two dosing regimens**. 90% prediction intervals and medians of simulated HbF% with HU 7/7 (solid lines) and 5/7 (dotted lines) (n = 10000).

**Figure 6:**
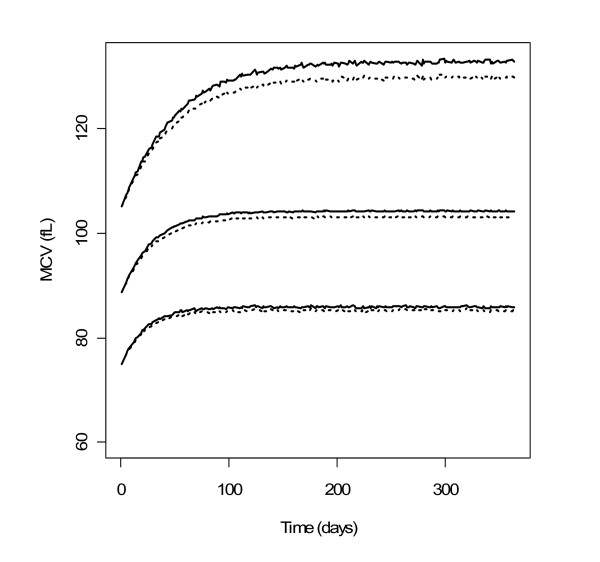
**Profiles of simulated MCV with two dosing regimens**. 90% prediction intervals and medians of simulated MCV with HU 7/7 (solid lines) and 5/7 (dotted lines) (n = 10000).

The simulated HbF% and MCV profiles with the two dosing regimens are shown in Figures 5 and 6 respectively. For MCV, the difference was very small. For HbF%, continuous dosing led to more significantly stronger response, especially for patients reaching the highest levels of HbF%. It can be observed that HbF% required a much longer time than MCV to reach the steady-state (approximately 26 and 3 months for 95% of steady-state levels respectively). The inter-individual variability of steady-state of HbF% was higher than that of MCV: the ratios of the 95^th ^to the 5^th ^percentile were approximately 10 and 1.5 respectively.

## Discussion

In this study, population PK-PD models were developed for the first time, in order to characterize the exposure-efficacy relationships of HU and its variability.

The pharmacokinetics of HU was found to be linear. Other studies in rats and in humans using doses ranging between 10 to 800 mg/kg in patients with malignancies identified parallel linear renal and saturable non-renal elimination [20]. The latter could not be detected in the studies reported here, probably because the doses administered were not high enough to reach saturation of non-renal elimination pathways (10 to 35 mg/kg *per os *in SCA). Otherwise, the presented model was consistent with the results of previously reported studies.

For a given dosing regimen, HU exposure varied approximately fivefold among patients. Part of the variability of apparent clearance and apparent volume of the central compartment was related to body weight. Clearance was better correlated with body weight at a 0.75 power, according to allometric scaling laws [21]. Because the maintenance dose of a drug to reach a desired average concentration is determined solely by its clearance, this allometric relationship implies that the HU dosing rate should be calculated with respect to body weight to the power of 0.75, or equivalently, to free fatty mass [22], in order to decrease the interindividual variability in HU exposure. Finally, the time to reach 95% of the pharmacokinetic steady-state was typically 35 days, in contrast with the delay to reach the maximal effect of HU, as discussed below.

The haematological results obtained in this study are compatible with those previously reported [23]. Our study brought further insight on the relationship between exposure and efficacy.

First, from a kinetic point of view, if we expressed the estimated K_out _parameters as half-lives and then multiplied them by 5 to obtain approximate times to reach steady-state before the drug is taken, we could see that they are around 265 and 83 days for HbF% and MCV respectively. HU is assumed to reduce K_out _and therefore extend this time to steady-state. The simulations under a constant dosing rate at 1000 mg per day show that 95% of the steady-state levels of HbF% and MCV need 26 months and 3 months to be reached, respectively. If the dosing regimen is modified, the same delay is required to reach a new steady-state. Hence, the variation of MCV is more rapid than that of HbF%. The 3 month delay for MCV is certainly related to the life span of RBC of 120 days and corresponds to the time needed to renew three quarters of RBCs.

Second, the effect of HU on HbF% was estimated to be at its maximum independently of the exposure, in the dose range of our study (500 to 2000 mg/day). However, the intensity of the effect (I_max_) varied among patients, with a typical value of 0.57 and a coefficient of variation of 27%. None of the demographic and biological indices was correlated with these variations. Part of this variability might be explained by genetic polymorphisms in genes regulating HU metabolism or transporters, HbF expression and erythroid progenitor proliferation [8-10]. These polymorphisms might modulate the patient response to HU. In addition, the HU-inducible small guanosine triphosphate-binding protein, secretion-associated and RAS-related (SAR) protein has been demonstrated to play a key role in *HBG *induction and erythroid maturation by causing cell apoptosis and G1/S-phase arrest [24]. Some genetic polymorphisms related to this pathway have been described such as *sar1a *promoter polymorphisms [10] and may also contribute to variability. Finally, patient compliance to treatment might also be a source of variability in response, but no information on compliance was available in this study.

Third, HU increased HbF% by reducing HbF elimination rate constant by 57% (for a typical patient). Absolute values of HbF per RBC (medians) at baseline and after 6 months were 1.9 pg and 5.6 pg respectively, which confirms that HU leads to a real increase in HbF per cell. Theoretically, a full inhibitor could reduce the elimination rate further, leading to a higher increase of HbF%. Hence, there is room for improvement, e.g. by looking for stronger inhibitors, or combining HU with other drugs to be discovered.

Fourth, a relation between HU exposure and effect on MCV could be identified, but this relation was flat as in the studied range of drug exposure the effect was close to its maximum. When the average HU concentration was 2 or 9 mg/L (the extremes of this study), the MCV decay rate constant (K_out_) was multiplied by 0.88 or 0.84 respectively, with an interindividual coefficient of variation of 49%. Hence the inhibition of MCV "elimination" by HU was less potent than that of HbF, and the interindividual variability was greater.

Regarding simulations, a close inspection of Figures 5 and 6 reveals that the interindividual variability of the steady-state values of HbF% and MCV are different, the ratio of the 95^th ^to 5^th ^percentile being approximately 10 and 1.5 respectively. Although the effects of HU on MCV and HbF% variations are correlated, the steady-state value of MCV at month 3 is not predictive of the HbF% value at month 26. Hence, HbF% level, which is also directly related to the relief of sickle cell disease symptoms, may be the best biomarker for monitoring HU treatment.

No dose-limiting toxicity occurred in this study, which prevented a toxicity model from being developed. Nevertheless, cytopenia may occur during HU treatment, leading to dose reduction. We compared by simulation two dosing regimens, one continuous daily and the other with interruptions of 2 days after every 5 days. The difference was very small regarding the MCV profile, but larger for the HbF% profile, particularly for simulated patients in the last quartile of HbF% distribution. For these patients, continuous dosing may induce a clinically relevant increase of HbF% compared with the discontinuous schedule. The limits of this simulation exercise are that genetic polymorphisms were not accounted for, and some other biomarkers (arginase, NO enzymes, activated adhesion molecules, phosphatidylserine externalization [25,26]) were not evaluated.

## Conclusions

The mode of action of HU on two clinically relevant biomarkers of its efficacy was established. The high variability of response to HU was related in part to pharmacokinetics (HU exposure varied approximately fivefold among patients), and to pharmacodynamics. The steady-state of HbF% and MCV levels need 26 months and 3 months to be reached, respectively, and the interindividual variability of the steady-state values of HbF% is much greater than that of MCV. As a result, the steady-state value of MCV at month 3 is not predictive of the HbF% value at month 26. Hence, HbF% level may be a better biomarker than MCV for monitoring HU treatment. Simulations showed that continuous dosing led to a stronger response than intermittent dosing (5 days out of 7), especially for patients reaching the highest levels of HbF%. Hence, a continuous dosing should be prescribed. Finally, an exciting perspective suggested by the model is that HbF could be further increased by more potent drugs or by drug combinations. In future studies, the model may allow to describe quantitatively the impact of relevant polymorphisms on the variability of response to HU, in order to refine the simulations and to yield specific recommendations for each genotype or haplotype.

## Competing interests

The authors declare that they have no competing interests.

## Authors' contributions

Study design: MT, AHulin and FG; inclusion and follow-up of patients: AHabibi, DB, FG; measurement of HU, monitoring of biological data: HS, KPDP and AHulin; statistical analysis, modelling and simulations: IP, PG and MT, writing of the manuscript: IP, MT, PG, AHulin and FG. All authors read and approved the final manuscript.
